# Repeat Percutaneous Radiofrequency Ablation of T1 Renal Cell Carcinomas is Safe in Patients with Von Hippel–Lindau Disease

**DOI:** 10.1007/s00270-021-02935-w

**Published:** 2021-08-19

**Authors:** Joel Wessendorf, Alexander König, Hendrik Heers, Andreas H. Mahnken

**Affiliations:** 1 Department of Diagnostic and Interventional Radiology, Marburg University Hospital, D, Philipps University Marburg, Baldingerstrasse, 35043 Marburg, Germany; 2 Department of Urology, Marburg University Hospital, D, Baldingerstrasse, 35043 Marburg, Germany

**Keywords:** Von Hippel–Lindau disease, Renal cell carcinoma, Renal tumor, Renal mass, Radiofrequency ablation

## Abstract

**Purpose:**

Patients with Von Hippel-Lindau disease often develop multifocal, metachronous renal cell carcinomas which require therapy. The purpose of this retrospective single-center study is to evaluate the outcomes of radiofrequency ablation (RFA) in the treatment of renal cell carcinomas in patients with Von Hippel-Lindau disease.

**Materials and Methods:**

9 patients (4 male, 5 female, 47.9 ± 10.7 y/o) with Von Hippel-Lindau disease underwent 18 CT-guided percutaneous RFA procedures for the treatment 21 renal cell carcinomas (largest diameter: 32.9 ± 8.6 mm, cT1a: 16, cT1b: 5). Seven patients were previously treated either by partial or radical nephrectomy. Technical success, effectiveness, safety, progression-free survival, overall survival and tumor characteristics were analyzed.

**Results:**

All RFA procedures were technically successful without major complications. There were 5 minor complications. No residual or recurrent tumor was seen in the ablation zone during a follow-up of 34.0 ± 18.1 months (0–58 months). No patient required dialysis during follow-up. One patient died after 63 months after the first treatment due to complications from a cerebellar hemangioblastoma. No endpoint was reached for overall or progression-free survival.

**Conclusions:**

The results from this limited case series suggest that RFA of RCCs in patients with VHL is a safe and effective therapy, which can preserve sufficient renal function even after renal surgery.

## Introduction

Patients with Von Hippel–Lindau disease (VHL) are predisposed to hereditary renal cell carcinomas (RCC) to which their mortality and morbidity are closely related [1]. These hereditary RCCs are known for multifocal syn- and metachronous development [2–4]. These characteristics set hereditary RCCs in VHL patients apart from sporadic RCCs.

Due to the special characteristics of RCCs in VHL patients, tumor control aims to prevent metastatic disease, which is unlikely to occur in tumors < 3 cm [5]. The main goals for VHL patients are tumor control and preservation of renal function. Both need to be achieved throughout multiple treatments.

Therefore, VHL patients need a repeatable nephron-sparing technique to achieve tumor control. Currently, there is a disparity between urological guidelines, which suggest partial nephrectomy (PN) [6, 7] and studies reporting promising outcomes for thermal ablations [8–12].

The aim of this study is to evaluate the oncologic outcome and renal function after RFA in the therapy of cT1 RCCs in VHL patients.

## Patients and Methods

Data of all patients diagnosed with VHL who underwent RFA for RCCs in a single tertiary referral center were anonymized and retrospectively evaluated.

Nine patients (4 male; 5 female) with 21 RCCs (largest diameter: 32.9 ± 8.6 mm, cT1a: 16, cT1b: 5) were treated during 18 RFA procedures (Table 1). Indications for therapy were either a tumor growth > 0.5 cm/ year or a tumor diameter > 3 cm. Prior to therapy tumor diameters and TNM score were assessed. Before the first ablation, the tumor number was counted, and the baseline eGFR was determined using the MDRD equation. Four tumors were embolized within 24 h prior to RFA with 250–700 μm microparticles and microcoils if needed. The indication to embolize was a case-by-case decision depending on tumor size and location. All ablations were performed under general anesthesia with non-fluoroscopic CT-guidance using posterolateral approach as previously described [13] (peak power output: 50–200 Watt; duration of energy deposition: 36.8 ± 17.9 min (10–83 min)). The used applicator was a LeVeen electrode (Boston Scientific Corp. Natick, MA) sized equal to the largest tumor diameter or slightly larger. Cold pyeloperfusion with saline solution (*n* = 6) and/or hydrodissection with a glucose solution (*n* = 6) were performed to protect the bowel or ureter near if closer than 5 mm to the target tumor.

**Table 1:** Patient characteristics (NSS = nephron-sparing surgery; RN = radical nephrectomy)

Patient	Age (years)	Surgery	Total tumors	Tumors treated / sessions	TNM stage	Oncologic outcome	Imaging follow-up (months)	eGFR change	Functional follow-up (months)
1	36	–	2	2/2	2 × cT1a	Tumor control	20	− 22,18%	74
2	45	NSS	12	3/3	1 × cT1a, 2 × Tc1b	Tumor control	54	− 39,72%	54
3	45	–	2	2/1	2 × cT1a	Tumor control	36	–	–
4	62	NSS	6	2/2	2 × cT1a	Tumor control	48	− 36,47%	49
5	29	NSS	19	5/4	3 × cT1a, 2 × Tc1b	Tumor control	58	− 79,06%	57
6	61	RN	2	2/2	2 × cT1a	Tumor control	27	− 47,20%	27
7	49	NSS	1	1/1	1 × cT1a	Tumor control	0	− 0,41%	12
8	51	NSS	3	2/1	2 × cT1a	Tumor control	35	− 22,39%	22
9	53	NSS	5	2/2	1 × cT1a, 1 × Tc1b	Tumor control	28	− 15,39%	21

The day after ablation cross-sectional imaging was performed to evaluate success of ablation, complications, the ablation zone and ablative margin. The ablation was considered complete if contrast enhancement was absent in the area of the former tumor. After one ablation, no contrast agent was used due to chronic kidney disease. Instead, protein denaturation in MRI was used to conclude complete ablation. Complications were assessed using the CIRSE classification of complications [14], the SIR classification system for complications by outcome [15] and the Clavien–Dindo classification [16]. Ablation zone size was measured by determining the three largest diameters that are perpendicular to each other, and ablative margin was calculated using tumor diameters and ablation zone diameters.

Further follow-up imaging included a contrast-enhanced CT or MRI scan 4 to 6 months after ablation and every 6 months thereafter.

The paired two-sample Student's t test was performed to analyze eGFR. A *p* value < 0.05 was considered statistically significant. Statistics were computed using IBM SPSS Statistics 27 (International Business Machines Corporation, Armonk, NY, the USA).

## Results

All target tumors were successfully ablated during the initial procedure. Technical success rate and technique effectiveness rate were both 100% with a minimum ablative margin of 1.0 mm. There was no residual or recurrent tumor in the ablated area during an imaging follow-up of 34.0 ± 18.1 months (0–58 months). Follow-up imaging regularly showed involution of the ablation zone. One patient died 63 months after the first treatment due to complications of a cerebellar hemangioblastoma.

Post-procedural laboratory parameters of one patient were not available, so the patient was excluded from the analysis of functional outcome. No patient required dialysis during follow-up. The mean eGFR declined over 39.5 ± 21.9 months (12–74 months) by -32.9 ± 23.9% from 65.7 ± 20.7 to 46.5 ± 25.3 ml/min (*P* < 0.05).

Five (27.8%) minor complications and no major complication occurred (Table 2). No complication prolonged the hospitalization or affected the treatment result. The hematuria and small urine leak were caused by a Mono-J-catheter insertion for retrograde pyeloperfusion in preparation for RFA.

**Table 2:** Types and treatment of procedures-related complications

Complication	CIRSE classification	SIR classification	Clavien–Dindo classification	Therapy
Asymptomatic pneumothorax	1	Minor, A	1	No therapy
Urinary bladder tamponade and hematuria	3	Minor, B	1	Irrigation catheter
Hematuria and urine leak in preparation for RFA	3	Minor, B	1	Irrigation catheter
Urinary tract infection and hematuria	3	Minor, B	2	Cefuroxim 250 mg
Skin burn	3	Minor, B	3	Excision and suture

In all ablations, the post-procedurally assessed ablation zone contained the entire target tumor. The minimum ablative margin size was 1.0 mm. Further follow-up imaging regularly showed involution of ablation zone.

## Discussion

The presented data illustrate that repeated thermal ablation is well suited to control renal tumors in VHL patients, while preserving sufficient renal function and avoiding dialysis. The oncologic outcome of this case series is consistent with previously published data [8–12]. Furthermore, this study displays RFA´s ability to achieve tumor control in larger tumors than reported previously in VHL patients, where lesion size ranged from 1.9 to 2.6 cm [8–11].

The complication rates of 0% major complications and 28% minor complications compare favorably to previously published complication rates of up to 8% major complications and up to 66% minor complications [8, 9]. Although Wei et al. [17] concluded no significant difference in overall complication rates between RFA and non-repeat nephron-sparing surgery (NSS), it is known that repeat and salvage NSS are more complicated than the initial NSS and associated with a higher risk for complications including loss of the treated kidney [18]. Therefore, renal ablation appears to be a safe technique and particularly well suited for repeated use.

Despite eGFR decrease sufficient preservation of renal function was achieved in every patient, especially considering chronic kidney disease in 3/9 patients prior to therapy and history of renal surgery in 7/9 patients. No patient became dependent on dialysis during follow-up. Data on renal function are not comparable to previous reports on renal RFA, as they evaluated the change in eGFR between the initial RFA and the last RFA. This study provides data on the long-term eGFR change. In studies with similar tumor sizes, a better preservation of renal function was observed after RFA, when compared to NSS [17, 19].

A unique feature of this study is the information regarding the ablative margin after renal RFA, whose recommended size is currently not agreed upon. In this study, oncologic control was achieved with a mean ablative margin below the recommended ablative margin for hepatocellular carcinomas, which is a minimum of 5 mm [20]. With preservation of renal function as a main goal in the treatment of hereditary RCCs, a small ablative margin was intended in all procedures. Considering the oncologic outcome in this case series, an ablative margin of ≥ 5 mm might not be necessary in the therapy of RCCs in VHL patients.

Thermal ablation compares favorably to the current gold standard of NSS as it is better suited to preserve renal function and excellent safety, even when performed repeatedly. When compared with the outcomes of cohorts with similar tumor size and comorbidities, it has no significant disadvantage in local tumor control and survival [11, 17–19].

Like previous reports, this study is limited by the low number of patients (range in the literature 6–14 patients) [8–11]. However, this case series provides information on a relevant number of tumors and procedures.

In conclusion, physicians should consider thermal ablation in the therapy of hereditary RCCs in VHL patients.
